# Science and Policy: Understanding the Role of Value Judgments

**DOI:** 10.1289/ehp.122-A192

**Published:** 2014-07-01

**Authors:** Janet L. Pelley

**Affiliations:** Janet L. Pelley, MS, based in Toronto, ON, Canada, writes for *Chemical & Engineering News* and *Frontiers in Ecology and the Environment*.

Scientists increasingly find themselves at the center of contentious public policy debates over issues such as chemical regulation and climate change. The general public and politicians often expect scientific advisors to be purely objective.^1^ However, a new commentary in this issue of *EHP* asserts that scientific research is intrinsically influenced by value judgments and that researchers should declare their interests and values as the best way to promote objectivity, transparency, public trust, and good policy.^2^

The authors wrote the commentary after observing the uproar last year following a preliminary European Commission (EC) review of its policy on endocrine-disrupting compounds.^3^ Eighteen prominent scientists published an editorial charging that the EC report was based on flawed reasoning.^4^ More than 70 researchers responded in a series of commentaries, calling in some cases for a clearer separation between science and policy.^5^^,^^6^^,^^7^

**Figure:**
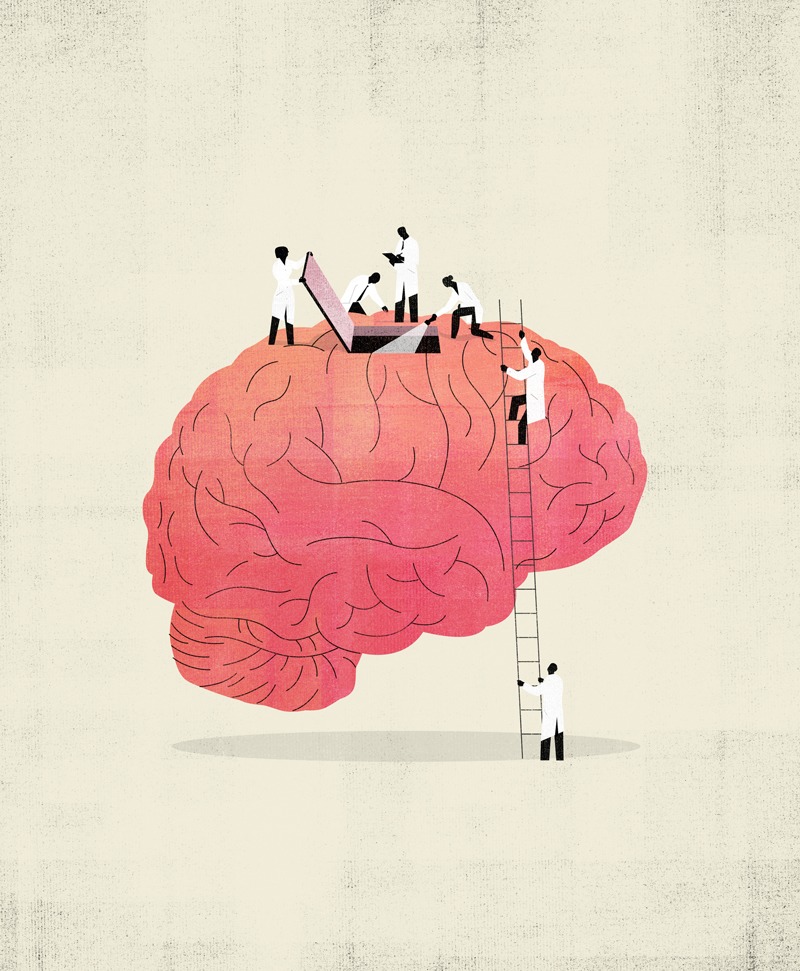
According to a new commentary, value judgments aren’t the problem; lack of transparency is. © 2014 Keith Negley c/o theispot.com

“Looking beyond the scientific details, the debate suggested that scientists should eliminate all interests and values from their research,” says lead study author Kevin Elliott, a professor of philosophy at Michigan State University. But a growing body of research backs up centuries of philosophical writings on human nature: “Values do affect people’s research,” Elliott says, “and there are benefits to being transparent about these values.”

Elliott and coauthor David Resnik, a bioethicist at the National Institute of Environmental Health Sciences, decided to use the conflict over the EC review to illustrate how personal ideals are embedded in the very nature of research itself. They explain that researchers’ values inform their presumptions. For instance, one point of contention over the EC review was whether evidence of toxicity in animals should be presumed to predict human effects in the absence of evidence to the contrary.

Investigators further draw on their values when they choose the standard of evidence upon which to base health-relevant policy—for instance, whether animal data alone are sufficient to make decisions, or whether animal and human data are required. In both cases, the choice of which evidence to use hinges on values about what kind of risks are acceptable, not science alone, Elliott says.

Critics of the EC review also debated whether endocrine-disrupting compounds have a threshold concentration below which no health effects are seen. “Underlying this dispute are value judgments about how much evidence is required to accept or reject a hypothesis,” Elliott says. Toxicologists traditionally work with the threshold hypothesis and require a lot of evidence before they reject it, he says. “Meanwhile, endocrinologists are not as entrenched in toxicological paradigms and therefore may not demand as much evidence before abandoning the threshold hypothesis.”

“Many scientific disagreements boil down to issues that are more at the normative end of the spectrum than the factual end, and this controversy over endocrine disruptors is a perfect example,” says Scott Findlay, an environmental scientist at the University of Ottawa. He agrees with Elliott and Resnik that policy-relevant scientific debates would be more productive and transparent if scientists disclosed their presumptions upfront, disclosed conflicts of interest, and clarified the pros and cons of multiple interpretations of the science.

“The advantage of discussing values is that people can start thinking about the fact that they make choices based on these internal aspects, and they can become more aware of those and minimize biases in research,” says James Kehrer, a toxicologist at the University of Alberta. But he sees problems with revealing more than financial and employment-related conflicts of interest. “There is no reasonable way to get at one’s core values without violating privacy,” he says.

Findlay points out that discussions about presumptions and standards of evidence could make scientists realize that what they thought was a scientific controversy in fact has little or nothing to do with science. “And if it has little or nothing to do with science,” he says, “then scientists are in no more of a privileged position to render an opinion than anyone else.”

## References

[1] Douglas HE. Science, Policy, and the Value-Free Ideal. Pittsburgh, PA:University of Pittsburgh Press (2009). Available: http://goo.gl/1794Hn [accessed 11 June 2014].

[2] Elliott KC Resnik DB Science, policy, and the transparency of values. Environ Health Perspect 122 7 647 650 2014; 10.1289/ehp.1408107 24667564 PMC4080531

[3] Cressey D Journal editors trade blows over toxicology. Nature News 20 September 2013; 10.1038/nature.2013.13787

[4] Dietrich DR Scientifically unfounded precaution drives European Commission’s recommendations on EDC regulation, while defying common sense, well-established science and risk assessment principles. Food Chem Toxicol 62 A1 A4 2013; 10.1016/j.fct.2013.07.005 23835284

[5] Bergman Å Science and policy on endocrine disrupters must not be mixed: a reply to a “common sense” intervention by toxicology journal editors. Environ Health 12 1 69 2013; 10.1186/1476-069X-12-69 23981490 PMC3765603

[6] Gore AC Policy decisions on endocrine disruptors should be based on science across disciplines: a response to Dietrich et al. Endocrinol 154 11 3957 3960 2013; 10.1210/en.2013-1854 PMC5398595 24048095

[7] Grandjean P Ozonoff D Transparency and translation of science in a modern world. Environ Health 12 1 70 2013; 10.1186/1476-069X-12-70 23981514 PMC3765922

